# Identification of TYR gene variants associated with white coat color in Hanwoo cattle using whole-genome sequencing

**DOI:** 10.1186/s12864-025-12458-0

**Published:** 2026-01-05

**Authors:** Jeong Woen Shin, Yoonsik Kim, Seung Hwan Lee, Se Young Lee, Jae-Yeong Lee, Chan-Lan Kim, Mi-Ryung Park, Yeoung-Gyu Ko, Yoonji Chung

**Affiliations:** 1 Institute of Biotechnology, Chungnam National University, Daejeon, 34134 Republic of Korea; 2 Institute of Agricultural Science, Chungnam National University, Daejeon, 34134 Republic of Korea; 3 Division of Animal & Dairy Science, Chungnam National University, Daejeon, 34134 Korea; 4 Animal Genetic Resources Research Center, National Institute of Animal Science, RDA, Hamyang, 50000, Korea; 5 Animal Biotechnology Division, National Institute of Animal Science, Rural Development Administration, Wanju, 55365 Republic of Korea; 6 Dairy Science Division, National Institute of Animal Science, Rural Development Administration, Cheonan,, 31000 Republic of Korea

**Keywords:** White hanwoo cattle, Coat color, Genetic diversity, *TYR* gene, Missense variant

## Abstract

**Background:**

Hanwoo cattle, a native Korean breed, display diverse coat colors, with White Hanwoo being extremely rare. Understanding the genetic basis of white coat color is essential for preserving genetic diversity and uncovering pigmentation mechanisms. This study aims to explore genetic differences between White and Brown Hanwoo and identify coat color-associated variants through genome-wide association study.

**Results:**

Population structure analysis revealed clear genetic difference between the Brown Hanwoo (B×B) and White Hanwoo (W×W) groups. The W×W_White population exhibited the highest number of runs of homozygosity (ROH, *n* = 423) and the highest identity-by-descent (IBD) value (0.3547), suggesting reduced genetic diversity and potential inbreeding due to artificial selection. In contrast, the B×B_Brown group showed lower ROH (*n* = 78) and IBD (0.2956), indicating greater genetic variability. Genome-wide association study identified 3,482 SNPs significantly related to coat color. Notably, five missense variants were discovered in *TYR*, *FAT3*, *ENSBTAG00000051637*, and *SPTY2D1*, including a G-to-C substitution in exon 2 of the *TYR* gene. This mutation caused a glycine-to-arginine amino acid change, and structural modeling indicated a potential alteration in *TYR* protein conformation and hydrogen bonding, suggesting its involvement in melanin biosynthesis and coat color expression.

**Conclusion:**

This study highlights significant genomic differences between White and Brown Hanwoo populations, including reduced genetic diversity in White Hanwoo due to potential inbreeding. In addition, it confirms the *TYR* gene as a critical determinant of coat color through the identification of a functionally relevant missense variant. These findings provide valuable insights for Hanwoo breeding and conservation strategies and lay the foundation for further functional studies on pigmentation-related genes.

**Supplementary Information:**

The online version contains supplementary material available at 10.1186/s12864-025-12458-0.

## Background

Hanwoo cattle, a native Korean breed, has been integral to Korean agriculture for centuries [1]. Traditionally, it has been classified by coat color—brown, black, white, blue, and brindle (Chikso)—with each variant serving different agricultural purposes [1]. As Korea transitioned to industrialization, the focus shifted toward meat production, with particular emphasis on brown Hanwoo, which has become synonymous with Korean beef [1, 2]. Currently, five types of Hanwoo are prevalent in Korea: brown Hanwoo, Jeju black Hanwoo, black Hanwoo, brindle Hanwoo (Chikso), and white Hanwoo [3]. Among these, White Hanwoo is exceptionally rare, making it a valuable subject for studying coat color variation. Its rarity also underscores its potential for genetic conservation and selective breeding as a unique livestock resource [4].

The conservation and improvement of the Hanwoo breed rely heavily on the understanding of genetic traits, such as coat color, which is influenced by various pigments and genetic factors [5–7]. Coat color holds cultural and esthetic significance and is closely linked to economic traits in cattle [8]. For example, research has shown that coat color, a key breeding objective, correlates with bull robustness and economic performance across Hanwoo breeds, including Chikso, black, white, and brown Hanwoo. These insights highlight the potential industrial applications of coat color genetics and their role in conservation efforts [2]. Moreover, comparative studies on other cattle breeds, such as Angus, further showed the economic impact of coat color variations [9]. For example, research comparing black Angus and red Angus has shown that coat color variations can influence feed efficiency and carcass composition, indicating that the genetic factors underlying coat color may also influence economically significant traits [9]. These insights underscore how genetic research can not only improve livestock management and breeding but also advance more sustainable and profitable agricultural practices.

Animal coat color is determined by the interplay of melanin and other pigments, regulated by genetic factors such as *MC1R*, *ASIP*, and *TYRP1* [10–12]. These genes, extensively studied in equine coat color (Rieder, Taourit et al. 2001), are believed to have a similar function in cattle. Research on the tyrosinase (*TYR*) has been ongoing, with studies showing that a mutation in exon 2 of *TYR* is responsible for the white coat color in Hanwoo [13, 14]. Additionally, the *TYR* has been consistently linked to coat color across multiple cattle breeds [15, 16]. K Jung, Y Choi and S Kim [14] reported that darker coat colors in Hanwoo exhibited higher *TYR* expression levels, suggesting its more active role in melanin synthesis.

**Fig. 1:**
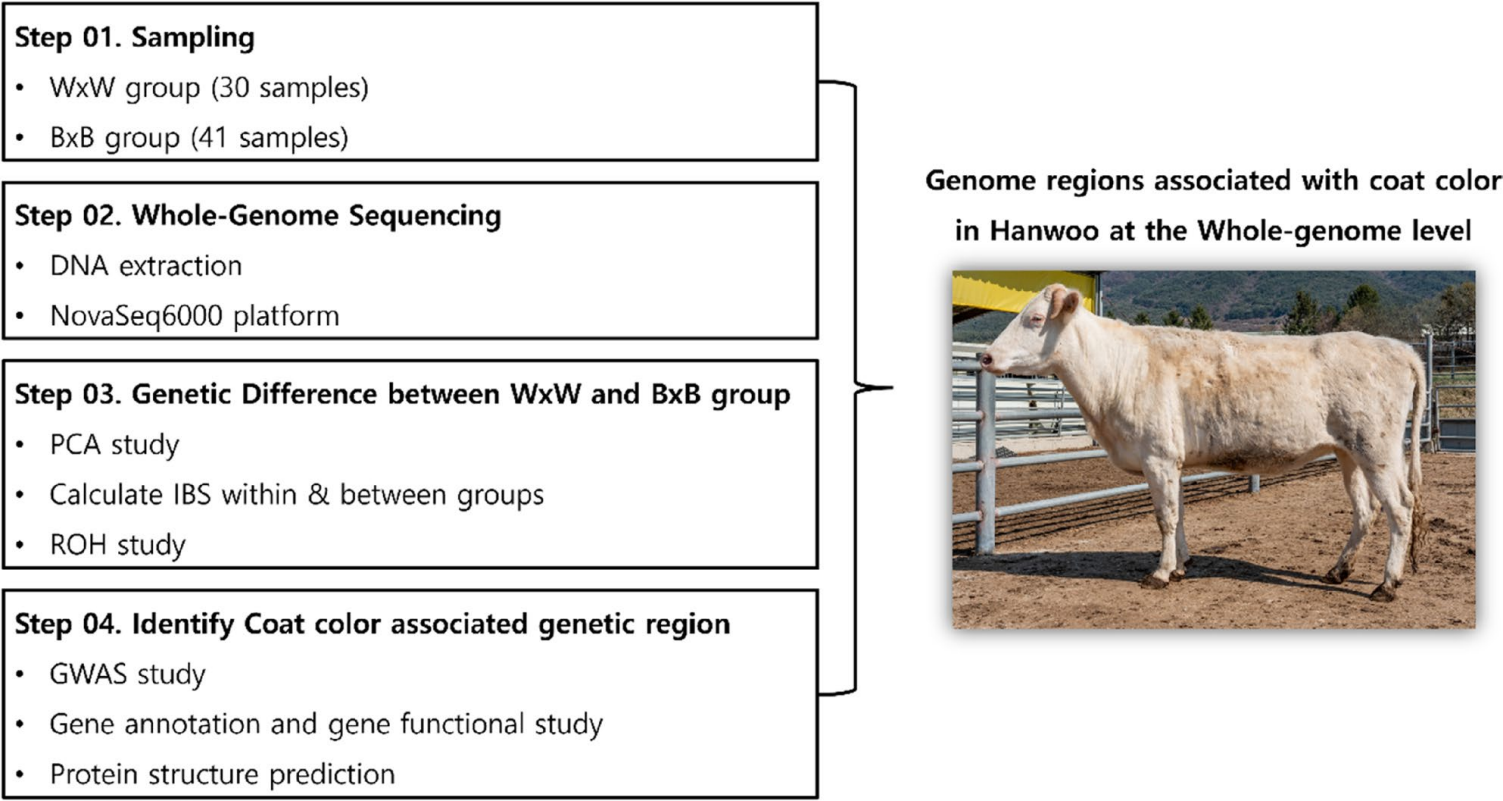
Graphical abstract. In this study, we used high-quality, whole-genome-level SNP data from White and Brown Hanwoo to assess genetic diversity between the two groups and to identify genomic regions significantly associated with coat color. Furthermore, among the coat color–associated variants, we evaluated missense variants by predicting their effects on protein structure to better understand their influence on phenotypic outcomes

In this study, we aimed to explore the genetic diversity underlying coat color in White Hanwoo and Brown Hanwoo populations and to identify genomic regions linked to coat color through a genome-wide association study (GWAS) using whole-genome SNP data. Additionally, we sought to examine the functional roles of genes within these regions and evaluate the structural impact of missense mutations in coat color-associated genes. Furthermore, we examined the impact of genotypic variations in these missense mutations on phenotypic differences, offering insights into the genetic mechanisms underlying coat color variations (Fig. 1).

## Methods

### Animals

In this study, the W × W group comprised a population with both parents being white Hanwoo cattle, while the B × B group consisted of a population with both parents being native brown Hanwoo cattle (Supplementary Figure S1). The study included 30 cattle from the W × W group and 41 from the B × B group. All cattle in the B × B group had brown (B × B_Brown), whereas the W × W group included 10 cattle with brown (W × W_Brown) coats and 20 with white (W × W_White) coat colors.

Sample collection was conducted by the National Livestock Genetics Resource Center of the Rural Development Administration in South Korea. All animal experiments and genotype data collection were conducted in compliance with the ethical standards set by the Institutional Animal Care and Use Committee of the National Institute of Animal Science, South Korea. Ethical approval was obtained before the study began, ensuring adherence to established guidelines and the humane treatment of all animals involved (protocol approved number: 2020 − 0444).

### High quality variant detection in whole-genome level

DNA was extracted from all samples, with concentrations ranging from 148.48 to 1,022 ng/µL. Libraries were prepared using the TruSeq Nano DNA kit and sequenced on the Illumina NovaSeq6000 platform to generate short-read whole-genome sequencing data. All experiments were conducted by TNT Research Co., Ltd. (http://www.tntresearch.co.kr/).

Raw sequencing data were subjected to quality control using FastQC (v0.12.1) and Trimmomatic (v0.39) to remove low-quality reads and adapter sequences (Bolger, Lohse et al. 2014). High-quality reads were then aligned to the ARS-UCD1.2 reference genome using BWA MEM (v0.7.17-r1188), followed by duplicate removal using GATK’s MarkDuplicates [17, 18]. Additional refinements included base quality score recalibration and synchronization of mate information.

Variants were called using GATK’s HaplotypeCaller, which generated GVCF files for all samples. These files were combined into a unified GenomicsDB for joint genotyping [17]. Final variant calling and consolidation were conducted using GenotypeGVCFs and GatherVcfs [17]. To ensure accuracy, stringent quality filters were applied, and high-quality SNPs were selected based on a genotype missing rate below 0.1 and a minor allele frequency of above 0.01, using PLINK1.9 (v1.90b5.2) [19].

### Genetic diversity analysis between W × W and B × B groups

To assess genetic differences between the W **×** W and B **×** B groups, population-level analyses were conducted. First, principal component analysis (PCA) was conducted using the “--pca” option in PLINK1.9 to examine genetic variation and structure [19].

To further explore genetic relationships and potential introgression events between the groups, a genomic relationship matrix (GRM) was constructed using GCTA (v1.94.1) [20]. The GRM enabled the quantification of genetic relatedness within and between groups, offering insights into the population structure. Additionally, the “--genome full” option in PLINK1.9 was used to calculate Identity by State (IBS) for all pairwise animal comparisons [19]. IBS values, which indicate the proportion of shared alleles regardless of inheritance, provided a detailed measure of genetic similarity across the groups.

To identify selection signals within each group, runs of homozygosity (ROH) were analyzed using the “--homozyg” option in PLINK1.9 [19]. ROH segments were defined as contiguous homozygous regions of at least 1 Mb.

All results were visualized using the “ggplot2” and “pheatmap” packages in R, facilitating insights into the genetic differences and selection patterns between the W **×** W and B **×** B groups [21, 22].

### GWAS by group and coat color

GWAS was conducted to identify genomic regions associated with coat color phenotypes, categorized as 0 (B **×** B_Brown), 1 (W **×** W_Brown), and 2 (W **×** W_White). The analysis was conducted using GCTA (v1.93.2) [20] based on a mixed linear model (MLM). In order to account for population structure and relatedness among individuals, a genomic relationship matrix (GRM) was included in the model as a random effect. To minimize the risk of Type I errors due to multiple testing, significant SNPs were identified using the Bonferroni correction threshold (*p* < 4.60 × 10^− 9^). In addition, previously reported genomic regions associated with coat color were highlighted in the Manhattan plot [23–32].

Functional annotation of the significant SNPs was performed using SnpEff 5.0 (build 2020-10−04 16:02) [33]. Additionally, minor allele frequency of the significant SNPs were calculated and compared across the three groups (B **×** B_Brown, W **×** W_Brown, W **×** W_White) using the “--freq” option in PLINK1.9 [19].

The GWAS results and minor allele frequency comparisons among the three groups were visualized using the “ggplot2” package in R [21].

### Gene function analysis and protein structure prediction

Gene function analysis was conducted using the Database for Annotation, Visualization, and Integrated Discovery to classify significant genes based on Gene Ontology (GO) terms with FDR < 0.05 [34]. These classifications included biological processes, molecular functions, and cellular components. AlphaFold2, with default parameters, was also used to predict protein structure changes resulting from missense variants in the coat color-associated gene [35, 36]. However, protein structure prediction was conducted only for the *TYR* variant due to its well-known functional relevance to coat color.

## Results

### High-quality variant discovery at the whole-genome level

The whole-genome sequencing of 73 samples generated approximately 679.70–774.73 million reads, yielding 102.60–116.80 Gb with a coverage depth of 33.10–37.68**×**. From these sequencing data, 24,138,535 genetic variants were identified, including 21,029,460 SNPs, which accounted for 87.12% of all detected variations. After applying quality control filters—excluding variants with a missing genotype ratio above 0.1 and a minor allele frequency below 0.01—10,870,738 high-quality SNPs remained for further analyses.

### Genomic structural differences between B × B and W × W groups

**Fig. 2:**
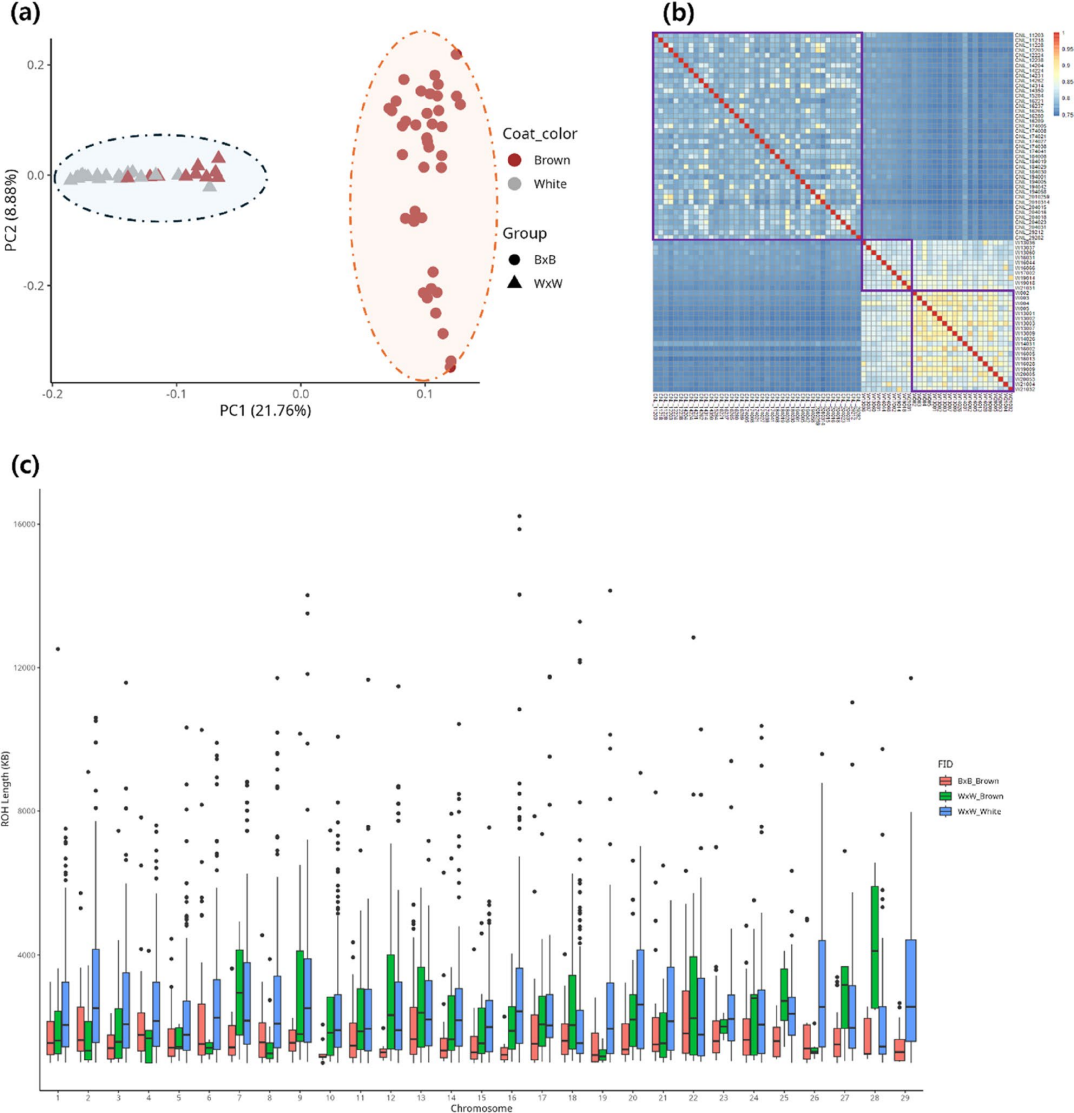
Results from a population analysis performed using a high-quality, whole-genome level SNPs. **a** Principal Component Analysis (PCA) results. The first and second principal components (PC1 on the x-axis and PC2 on the y-axis) explain 21.76% and 8.88% of the total variance, respectively. Each dot represents an individual sample, with coat color indicated by the dot’s color and group indicated by the dot’s shape. **b** Identity-by-state (IBS) analysis results. Pairwise similarity among individuals is represented by color, with blue indicating lower similarity and red indicating higher similarity. The groups are delineated by purple boxes, from left to right: B x B_Brown, W x W_Brown, and W x W_White. **c** Distribution of runs of homozygosity (ROH) longer than 1000 Kb in each group, shown by chromosome. ROH lengths are visualized with a boxplot, where the x-axis indicates the chromosome and the y-axis indicates ROH length

A PCA of 71 Hanwoo cattle revealed that Principal Component 1 (PC1) and Principal Component 2 (PC2) accounted for 21.76% and 14.70% of this genomic variance, respectively. The sample distribution based on eigenvectors for PC1 and PC2 revealed a distinct genetic separation between the B **×** B and W **×** W groups (Fig. 2a). Additionally, within the W **×** W, W × W_Brown and W × W_White group exhibited partial clustering; however, their separation was not well-defined.

Pairwise distances were calculated using minor and major allele combinations to assess genetic similarities within and between the three groups. The average IBS value was lowest in the B **×** B_Brown group (0.80) and highest in the W **×** W_White group (0.87). Notably, the mean IBS value between B **×** B_Brown and W **×** W_Brown was 0.01 higher than that between B **×** B_Brown and W **×** W_White, indicating greater genetic similarity between these groups (Table 1; Fig. 2b).

**Table 1:** IBS value between groups

Group1	Group2	Min	1st_Qu	Median	Mean	3rd_Qu	Max
BxB_Brown	BxB_Brown	0.7559	0.782	0.7889	0.7998	0.8003	1
WxW_Brown	WxW_Brown	0.8122	0.8205	0.8278	0.8474	0.8393	1
WxW_White	WxW_White	0.8023	0.8367	0.8574	0.866	0.8863	1
BxB_Brown	WxW_Brown	0.7545	0.7705	0.7749	0.7744	0.7783	0.7954
BxB_Brown	WxW_White	0.7467	0.7597	0.7632	0.7639	0.7671	0.7868
WxW_Brown	WxW_White	0.8049	0.8203	0.8316	0.8333	0.8392	0.9119

Furthermore, ROH regions > 1 Mb were identified in each group: 78 in B **×** B_Brown, 423 in W **×** W_White, and 3,294 in W **×** W_Brown (Table 2). Across all groups, ROH regions within the 1,000–2,000 Kb range were the most frequent. A detailed analysis of ROH length distribution, using 1,000-kb bin sizes, showed that W **×** W_White had a higher number of ROH regions across all lengths than the other groups. Specifically, the B **×** B_Brown group had 385 more ROH regions of 1,000–3,000 Kb than W **×** W_Brown but fewer ROH regions exceeding 5,000 Kb (Fig. 2c). Notably, very long ROH regions (13,000–16,000 Kb) were uniquely observed in the W **×** W_White group. When examining ROH distributions across chromosomes, the B **×** B_Brown group had shorter ROH lengths across most chromosomes than the other groups, while W **×** W_Brown showed no ROH regions on chromosome 29 (Fig. 2c).

**Table 2:** ROH length distribution

Group	ROH count	ROH Length														
		1000–2000	2000–3000	3000–4000	4000–5000	5000–6000	6000–7000	7000–8000	8000–9000	9000–10,000	10,000–11,000	11,000–12,000	12,000–13,000	13,000–14,000	14,000–15,000	15,000–16,000
BxB_Brown	782	558	140	46	15	12	6	2	2	0	1	0	0	0	0	0
WxW_Brown	423	229	84	46	34	10	9	6	1	1	1	0	2	0	0	0
WxW_White	3,294	1,570	745	410	236	141	54	57	37	16	11	9	2	2	3	1

### 3.5-K SNPs associated with coat color

**Fig. 3:**
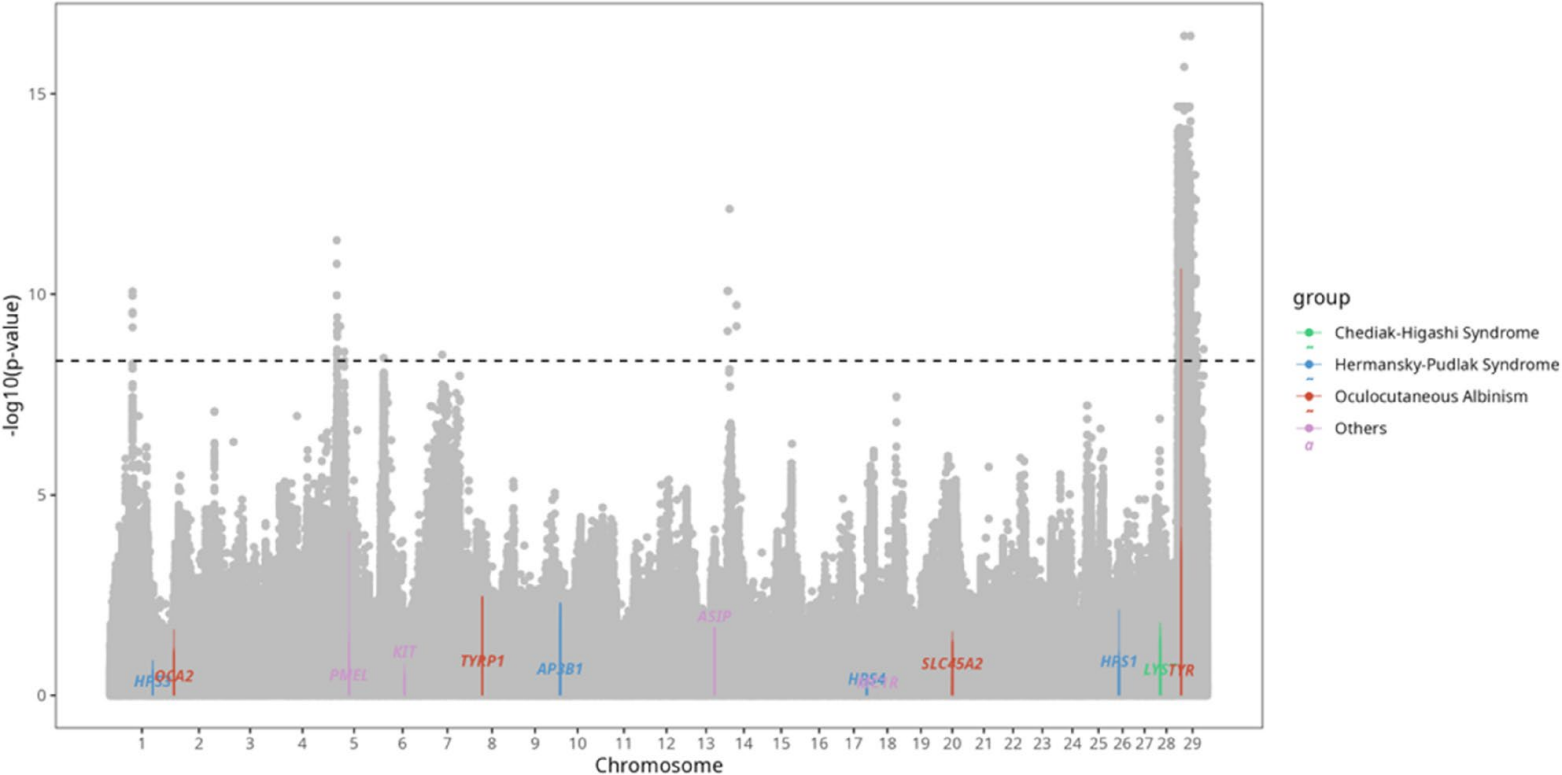
Genome-wide association study (GWAS) results for coat color. Each dot represents the p-value (transformed by -log10 for visualization) of the association between each SNP and coat color traits. Genes identified as being coat color–associated are shown in different colors according to the coat color–related biological processes they are involved in. The black dotted line indicates the Bonferroni significance threshold

**Fig. 4:**
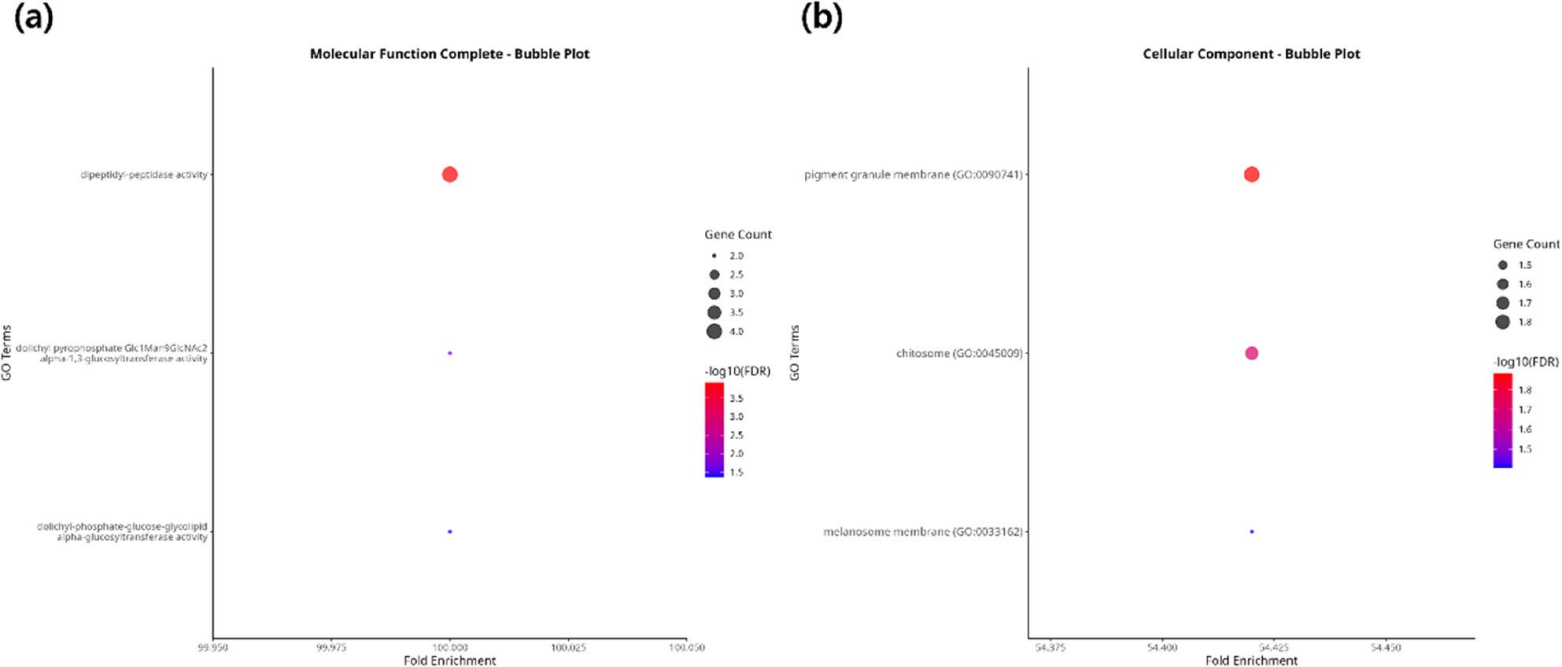
Gene Ontology results for genes harboring variants significantly associated with coat color. **a** Biological Process and **b** Cellular Component. In both panels, the x-axis shows fold enrichment and the y-axis lists the significant GO terms. More significant terms are indicated by deeper shades of red, and the dot size increases in proportion to the number of associated genes

A GWAS identified 3,482 SNPs significantly associated with coat color. These SNPs were distributed across chromosomes 1, 5, 6, 7, 14, and 29, with chromosome 29 containing the highest proportion (Fig. 3). They were associated with 89 genes and mapped to 162 transcripts. GO analysis revealed three significant molecular function terms—dolichyl-associated glucosyl transferase activity and dipeptidyl-peptidase activity—as well as three cellular component terms, all associated with the melanosome membrane, reinforcing the strong connection between these SNPs and coat color (Fig. 4a & b).

**Fig. 5:**
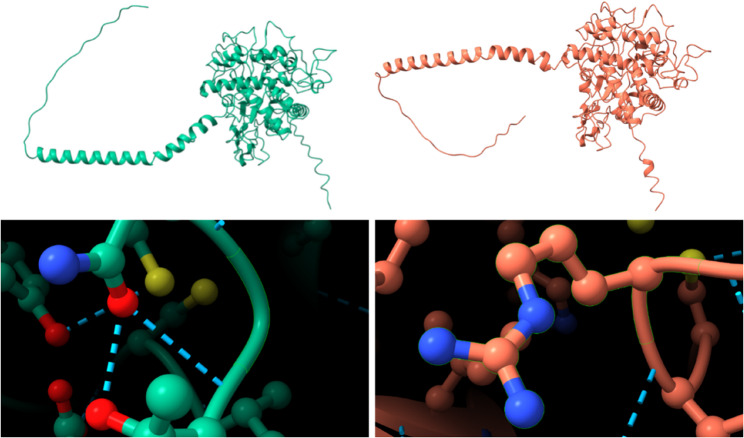
Predicted protein structures of the *TYR* with and without the missense variant. The structure on the left represents the wild-type protein, while the structure on the right shows the predicted structure containing the missense variant. The lower panels provide a more detailed view of these structures

Among these SNPs, five missense variants were identified in *FAT3*, *TYR*, *ENSBTAG00000051637*, and *SPTY2D1* (Table 3). Notably, *TYR* is well established as a key determinant of coat color. A significant mutation identified through GWAS in this gene involves a G-to-C allele change, resulting in a glycine to arginine substitution in the protein sequence. Structural analysis revealed significant differences in the 3D conformation of the mutant *TYR *proteins compared to the wild-type (Fig. 5). While the overall folding pattern of the mutant protein did not significantly differ from that of the wild-type protein, a closer examination of the magnified structure revealed notable changes in the hydrogen bonding patterns between specific amino acids.

**Table 3:** Annotation information for coat color related missense variants

SNP	Chromosome	Position	A1	A2	Frequency	*p*-value	Gene	Exon number	Protein coding
rs437603005	29	2,634,116	G	C	0.389706	3.49E-10	*FAT3*	1	p.Ser460Thr
29:6425026	29	6,425,026	T	C	0.367188	2.28E-11	*TYR*	2	p.Gly291Arg
rs383496439	29	17,828,834	A	G	0.328571	3.33E-12	*ENSBTAG00000051637*	2	p.Ala100Val
rs110633453	29	26,114,310	A	G	0.328571	2.98E-09	*SPTY2D1*	3	p.Arg212Gln
rs110690002	29	26,114,418	G	A	0.328571	2.98E-09	*SPTY2D1*	3	p.His248Arg

## Discussion

In this study, we aimed to identify genetic differences at the whole-genome level between White Hanwoo and Brown Hanwoo, a native Korean cattle breed. Additionally, we conducted a GWAS for coat color, followed by gene annotation and functional analysis of significant variants. Finally, we predicted the protein structures of coat color-associated genes to assess the potential impact of these genetic variations.

PCA analysis revealed a clear genetic distinction between the B × B and W × W groups. Notably, within the W × W group, cattle with brown and white coat colors exhibited partial clustering, although a complete separation was not observed. While their overall genetic background is similar, specific genomic regions may contribute to coat color differences. IBS analysis further showed that the genetic similarity between B × B_Brown and W × W_Brown groups was higher than between B × B_Brown and W × W_White groups, indicating that cattle with brown coat color in the W × W group share a closer genetic relationship with the B × B_Brown cattle. Additionally, the highest average IBS value (0.866) was observed within the W × W_White group, indicating a high degree of genetic homogeneity in this population.

The persistence of the white coat color trait was strongly influenced by specific genetic factors, as confirmed through PCA and IBS analyses. PCA results revealed that within the W × W group, brown and white cattle formed partial clusters but were not entirely distinct. Notably, brown W × W cattle clustered closely with B × B_Brown cattle, indicating a potential historical genetic relationship due to past crossbreeding. However, IBS results showed that the white coat color persisted despite crossbreeding with B × B_Brown. In particular, W × W_White exhibited a high IBS value (0.8049) with W × W_Brown cattle, indicating that the white coat color was naturally preserved within the W × W group. This implies that white cattle did not diverge independently but retained a close genetic relationship with other W × W group cattle. Conversely, the IBS value between white cattle and B × B_Brown cattle was relatively lower (0.7467), indicating a weaker direct genetic association between the two groups. The continued manifestation of the white coat color phenotype despite crossbreeding with B × B_Brown indicates that this trait was inherently present in the W × W group. Given these findings, the genotype responsible for the white coat color is likely recessive, meaning that when brown and white cattle are crossed, the offspring can exhibit both coat colors.

The W × W_White cattle exhibited reduced genetic diversity, likely influenced by artificial breeding. This group showed the highest number of ROH regions, with notably long ROH segments (13,000–16,000 Kb) observed exclusively within it. These long ROH segments reduce heterozygosity and increase homozygosity, thereby increasing the likelihood of allele fixation. Frequent inbreeding further diminishes genetic diversity by increasing the proportion of genes inherited from common ancestors within the population. Our IBD analysis also confirmed that the W × W_White population had the highest IBD value (0.3547), indicating a high degree of inbreeding and repeated artificial breeding (Supplementary Note 1). This is consistent with the history of backcrossing observed in this group. Conversely, the B × B_Brown group maintained relatively high genetic diversity, as confirmed through ROH and PCA analyses. This group belongs to the largest native Korean cattle population, where diverse breeding has preserved multiple lineages. Continuous genetic recombination has gradually shortened long ROH segments, allowing the population to maintain higher genetic diversity. Overall, these findings reveal distinct genetic structural differences between B × B and W × W groups, with the B × B_Brown group exhibiting greater genetic diversity than the W × W_White group. Furthermore, long ROH segments on chromosome 29 in the W × W_White group indicate that specific genomic regions within this chromosome have become fixed, potentially due to their association with coat color.

GWAS analysis identified 3,482 SNPs significantly associated with coat color, with the highest concentration on chromosome 29. GO enrichment study revealed significant terms related to melanosome membrane and pigment granule membrane in the cellular component category, and α-glucosyl transferase activity in the molecular function category. These terms are biologically relevant to melanogenesis, as melanosome membranes play a crucial role in melanin synthesis and enzyme trafficking [37]. In particular, α-glucosyl transferases are involved in the glycosylation and activation of key melanogenic enzymes [38]. Among the genes contributing to these terms, GPNMB, a melanosome-associated glycoprotein known to mediate pigment transfer between melanocytes and keratinocytes, was identified [39]. These results support the idea that genomic variation on chromosome 29 influences coat color by modulating melanosome structure and function.

Among the significant variants, five missense mutations were identified in *TYR*, *FAT3*, and *SPTY2D1*. The *TYR* is well known as a key determinant of coat color traits in cattle, and in this study, we observed significant GWAS signals within this gene [40, 41]. Notably, the identified variant is located in exon 2, a genomic region previously linked to coat color variation, further reinforcing its relevance [42]. This missense variant involves a G-to-C substitution, resulting in an amino acid change from glycine to arginine. Structural analysis indicates that this alteration may induce conformational changes in the *TYR* protein, potentially affecting its enzymatic function in melanin biosynthesis. Consequently, this genetic variation likely influences coat color phenotypes by modulating melanin production.

Although *FAT3* and *SPTY2D1* are not directly implicated in coat color trait, their significance in the GWAS results suggests the possibility of indirect regulatory effects or linkage signals. *FAT3* is involved in neural development and cell adhesion [43]. Given that melanocytes originate from neural crest cells, variations in *FAT3* may influence the migration, adhesion, or localization of these cells during embryonic development, which could in turn lead to subtle changes in melanocyte distribution and melanin deposition patterns [44–46]. Next, *SPTY2D1* encodes a histone chaperone protein related to chromatin remodeling [47]. Its role in transcriptional regulation suggest a potential influence on the expression of coat color-related genes such as *TYR*.

While this study provides meaningful insights into the genetic basis of coat color in Hanwoo, certain aspects could be further improved in future research. The relatively small sample size, particularly in the W×W_Brown group (*n* = 10), may have limited the statistical power to detect additional associations. In addition, coat color classification was based on expert visual assessment, which, although practical, may introduce some degree of subjectivity compared to standardized colorimetric methods. Protein structure prediction was performed for a key missense variant in the TYR gene; however, further validation through gene expression analysis or functional experiments could provide deeper insights into the biological impact of the identified variants. Future studies addressing these points with larger cohorts, objective phenotype measurements, and functional assays will be valuable in building upon the present findings and refining our understanding of coat color genetics in Hanwoo.

## Conclusions

In this study, we explored the genetic diversity and coat color-associated genetic traits of white and brown Hanwoo, offering essential insights into Hanwoo conservation and breeding strategies. By predicting the effects of missense variants in coat color-associated genes, such as *TYR*, this study enhances our understanding of the genetic mechanisms underlying coat color phenotypes. Future functional studies on these genes should support the improvement of economically significant traits and genetic conservation efforts. Additionally, further research incorporating gene expression analysis and functional validation will help clarify the precise mechanisms linking GWAS identified variants to coat color at the transcriptomic level. While this study provides valuable insights, further investigations with larger sample sizes and experimental validation will be beneficial to strengthen and expand upon these findings.

## Supplementary Information

Supplementary Material 1.

## Data Availability

The raw genomic data generated and analyzed in this study are not publicly available due to national livestock genetic resource protection regulations in Korea. These policies restrict public sharing of Hanwoo genomic sequences. Data access may be granted upon reasonable request to the corresponding author, pending approval from the National Institute of Animal Science (NIAS), Rural Development Administration, Republic of Korea. To support transparency and reproducibility, we provide summary statistics of the variants used in our analyses in Supplementary Figures S2–S3.
