# Supplementary material for: Identification of TYR gene variants associated with white coat color in Hanwoo cattle using whole-genome sequencing
Source: BMC Genomics. 2026 Jan 5;27:128. doi: 10.1186/s12864-025-12458-0 (PMC12870947; doi:10.1186/s12864-025-12458-0)
Supplement: Supplementary file 1 — Supplementary Material 1. [file 12864_2025_12458_MOESM1_ESM.docx]

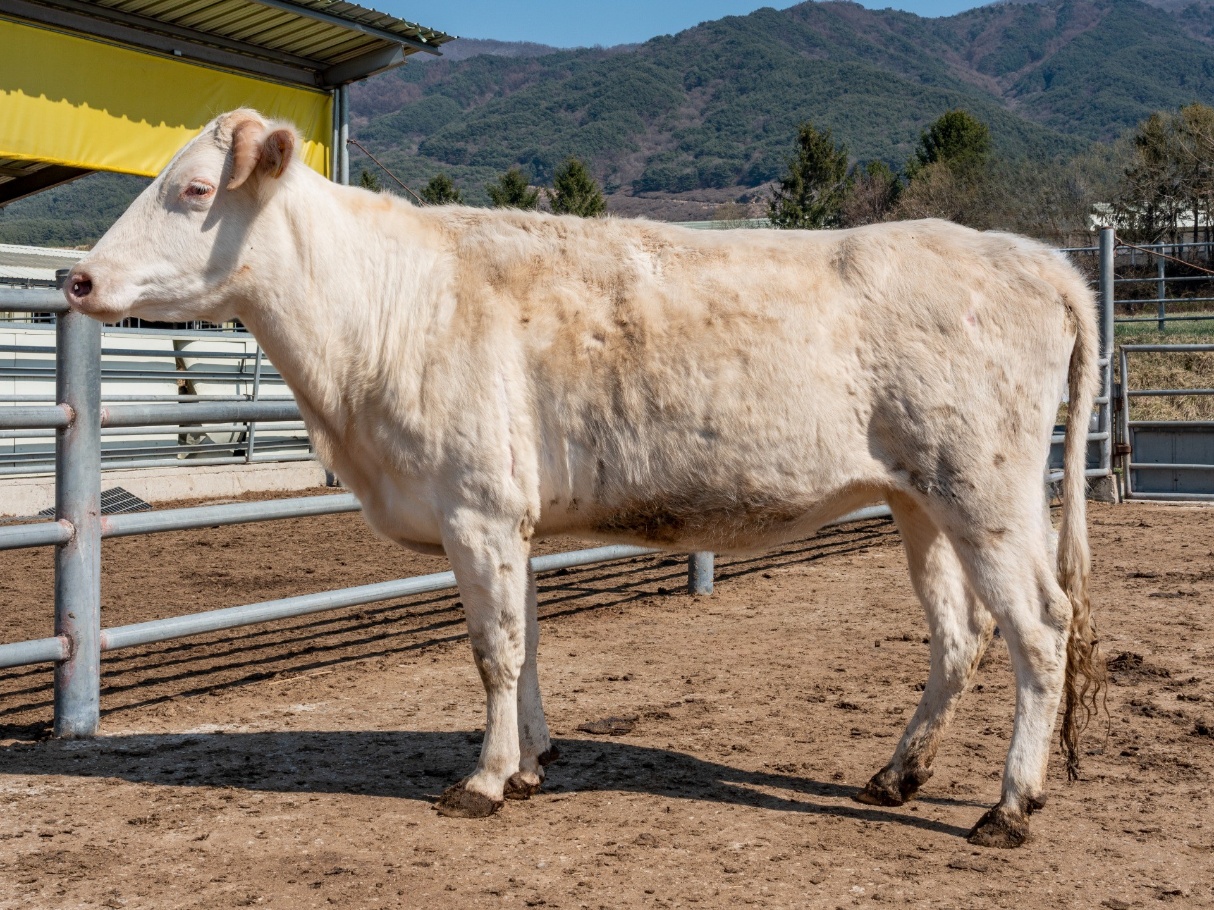
**Supplementary Figure S1. Coat color of white hanwoo**

**Supplementary Figure S2. SNP distribution across 29 chromosomes**


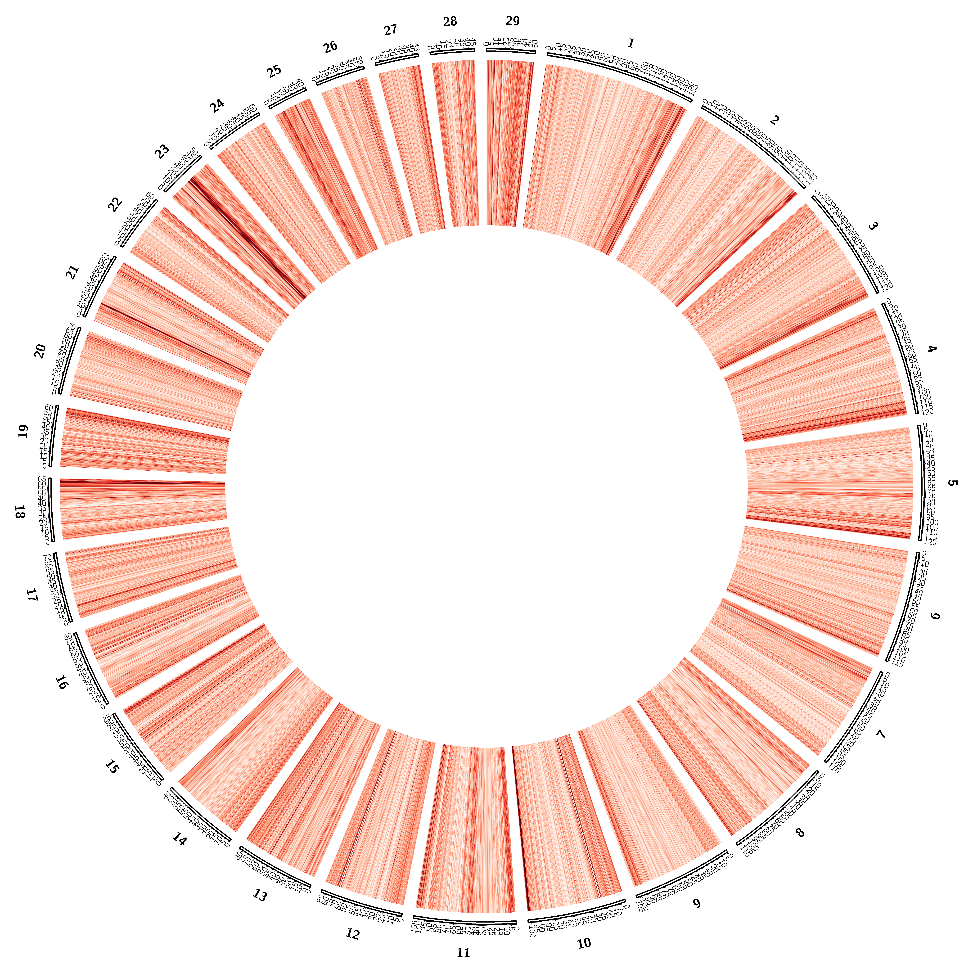


| **Chromosome** | **Count** | **Chromosome** | **Count** | **Chromosome** | **Count** |
| --- | --- | --- | --- | --- | --- |
| 1 | 629,486 | 11 | 466,994 | 21 | 327,582 |
| 2 | 501,635 | 12 | 377,091 | 22 | 252,477 |
| 3 | 510,545 | 13 | 407,109 | 23 | 326,958 |
| 4 | 526,955 | 14 | 343,949 | 24 | 268,384 |
| 5 | 496,108 | 15 | 394,249 | 25 | 247,948 |
| 6 | 463,368 | 16 | 357,741 | 26 | 228,759 |
| 7 | 425,537 | 17 | 328,029 | 27 | 209,216 |
| 8 | 460,019 | 18 | 339,264 | 28 | 224,407 |
| 9 | 391,464 | 19 | 325,009 | 29 | 291,272 |
| 10 | 451,599 | 20 | 297,584 |  |  |

This figure depicts the genome-wide distribution of SNPs across 29 chromosomes using a Circos plot. The bin size is 100 kb, and darker colors indicate higher SNP density. The table below summarizes the total number of SNPs per chromosome.

**Supplementary Figure S3. Minor allele frequency for SNPs**


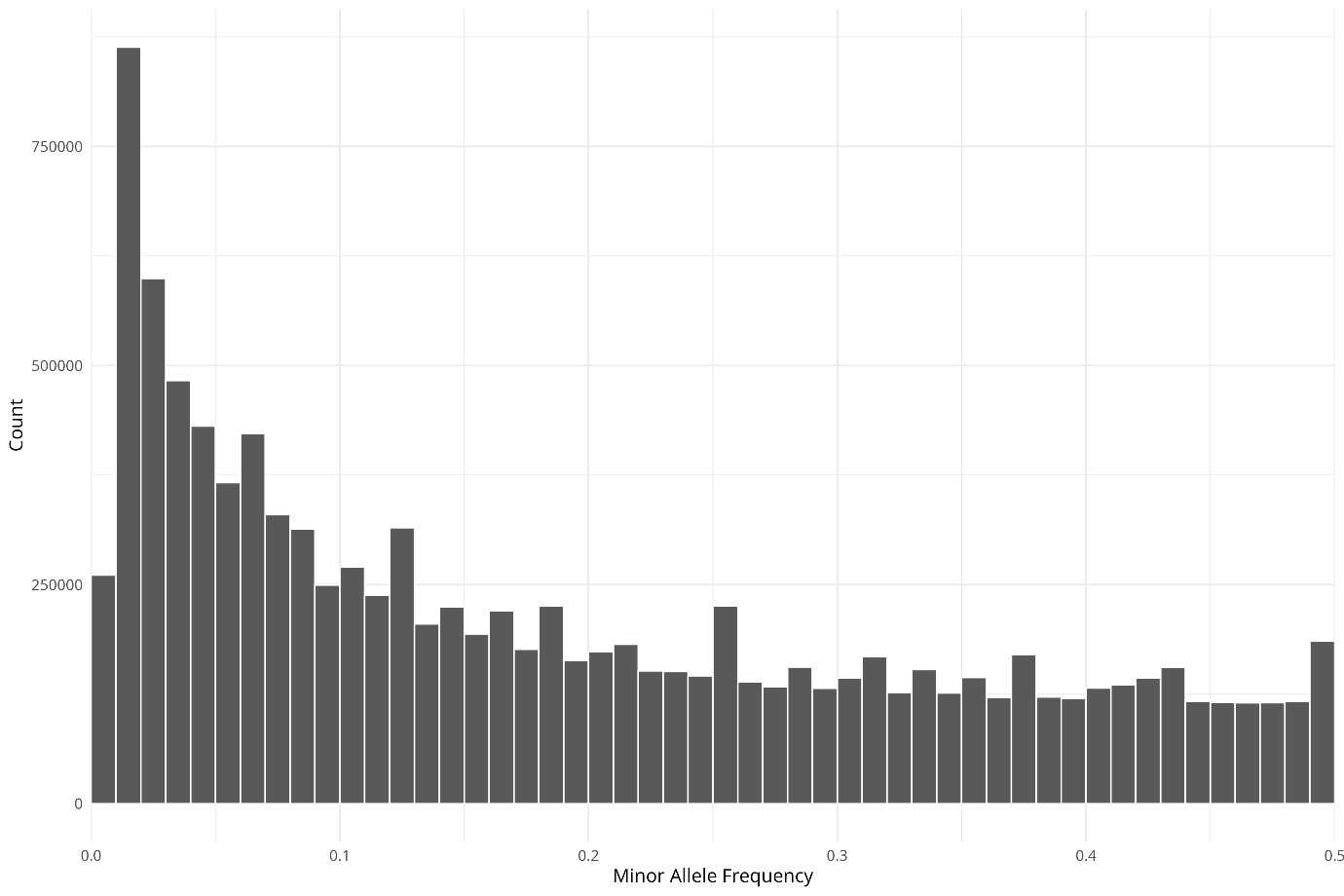


This figure shows the distribution of minor allele frequencies (MAF) for the SNPs used in the analysis. The bin size is 0.01; the x-axis is MAF, and the y-axis shows the number of SNPs per bin. The mean MAF is 0.18.
